# Assessment of Environmental Contamination With Soil‐Transmitted Helminth Eggs From Human and Animal Faeces in Southern Côte d′Ivoire: A Rapid Approach for Identifying High‐Risk Communities

**DOI:** 10.1155/japr/2672432

**Published:** 2026-07-24

**Authors:** Nadège Akissi Kouame, Jean Tenena Coulibaly, Jules N′Gatta Kouadio, Taki Jean Deles Avenie, Marie-Joëlle Alloua Aliali-Bedia, Sadikou Toure, Laurent Kouamé Valian, Kigbafori Dieudonné Silue

**Affiliations:** ^1^ Laboratoire de Biologie et Santé, UFR Biosciences, Université Félix Houphouët-Boigny, Abidjan, Côte d’Ivoire, univ-fhb.edu.ci; ^2^ Ecole Doctorale-Biologie Environnement et Santé (ED-BES), Université Félix Houphouët-Boigny, Abidjan, Côte d’Ivoire, univ-fhb.edu.ci; ^3^ Centre Suisse de Recherches Scientifiques en Côte d′Ivoire, Abidjan, Côte d’Ivoire, csrs.ch; ^4^ Swiss Tropical and Public Health Institute (Swiss TPH), Allschwil, Switzerland; ^5^ University of Basel, Basel, Switzerland, unibas.ch; ^6^ Centre de Santé Urbain de Irobo, Jacqueville, Côte d’Ivoire

**Keywords:** animals, Côte d′Ivoire, school-aged children, soil, soil-transmitted helminths, stagnant water

## Abstract

Soil‐transmitted helminth (STH) infections remain a major public health problem in low‐ and middle‐income regions, including the humid and lagoon areas in Côte d′Ivoire, where sanitation facilities are limited at the community level. Few studies have investigated the relationship between environmental (soil and water) contamination by STH eggs and their infection prevalence in humans and animals in endemic areas. We assume that assessing soil contamination with STH eggs could provide a rapid and cost‐effective indicator for identifying high‐risk communities. A cross‐sectional study was conducted in October 2024 in three villages (Attehou, Bekpou and Tiagba) selected based on previous epidemiological data. A total of 215 and 58 stool samples, respectively, from school‐aged children and animals (pigs, goats and sheep), in addition to 86 soil samples and 41 stagnant water samples, were collected. Human stool samples were examined using the Kato–Katz method, and animal stool samples using the McMaster flotation technique. Soil samples were analysed using an optimised flotation method with different solutions, and water samples using a sedimentation‐concentration technique. Proportions were compared using Fisher′s exact test. STH eggs were detected in all matrices investigated, with an overall prevalence of 44.6%, 24.1%, 25.5% and 2.43%, respectively, in children, animals, soil and water. Only *Ascaris*, *Trichuris* and hookworm eggs were identified. The highest prevalence was observed in Tiagba among children (83.67%) and soil samples (33.33%). NaCl solution provided better egg recovery from soil samples (> 50%, *p* = 0.02). Higher soil egg counts were observed in the same villages where higher egg counts were found in children, particularly in Attehou and Tiagba. The abundance of STH eggs in the environment was associated with that observed in school‐aged children. Our findings suggest that egg density in contaminated soil could serve as a reliable and cost‐effective alternative to human stool examination for identifying high‐risk communities, particularly in resource‐limited settings. Furthermore, assessing soil contamination could help guide integrated control strategies and epidemiological surveillance approaches aiming to reach eliminating STH infections.

## 1. Background

Soil‐transmitted helminths (STHs), including roundworms (*Ascaris lumbricoides*), whipworms (*Trichuris trichiura*) and hookworms (*Necator americanus*, *Ancylostoma duodenale* and *Ancylostoma ceylanicum*), infect humans and are transmitted through contaminated soil [1]. Other species belonging to these same genera infect livestock. Pigs are infected with *Ascaris suum* and *Trichuris suis* [2], whereas in small ruminants, *Trichuris ovis* infects both sheep and goats [3–5], and *Trichuris skrjabini* is found specifically in goats [6]. These parasites constitute a major public health concern in tropical and subtropical regions, primarily affecting disadvantaged populations living in poor conditions and exposed to inadequate sanitation and faecal waste management, including high‐risk practices such as open defecation [7, 8].

In Côte d′Ivoire, and particularly, in the humid and lagoon regions of the south, the prevalence of STHs remains high, driven by the suitable environmental conditions which are maintaining the parasites′ life cycle [9–11]. Several studies have shown that rural and periurban areas are worst affected, with infection rates up to above 50% among school‐aged children [10–12]. Exposure to contaminated soil, combined with limited access to safe drinking water and poor hygiene practices, contributes to maintaining transmission hotspots [13, 14]. This increases the risk of infection through both direct contact, such as walking barefoot or handling contaminated objects, and indirect contact via contaminated dust [15].

Attehou, Bekpou and Tiagba are rural communities located in southern Côte d′Ivoire, where hygiene conditions remain poor due to the widespread practice of open defecation [16]. In addition, most households lack access to safe drinking water [17, 18]. The playgrounds frequented by children are unsuitable, with stagnant water and proximity to excreta disposal sites, which may serve as potential sources of environmental contamination and infection.

Most studies on environmental contamination with STHs have focused on parasite species with zoonotic potential to assess the risk of transmission from animals to humans [19–22]. In contrast, few studies have investigated high‐risk sites for STH transmission, such as those recently conducted in Cameroon [23] and Kenya [24], where soil samples were collected from households [24, 25], latrines [23], playgrounds [14, 23] and markets [14]. These studies highlighted high contamination rates, respectively, for *Ascaris* (27%), *Trichuris* (77%) [14] and hookworm (27.5%). However, the diagnostic methodologies and sampling periods varied considerably across study settings, making comparisons difficult [13, 14, 25]. Therefore, this study is aimed at examining the relationship between environmental contamination (soil and water) by STH eggs and their prevalence in humans and animals to assess the feasibility of using environmental surveillance as a rapid and cost‐effective indicator for identifying high‐risk communities in endemic settings.

## 2. Material and Methods

### 2.1. Study Design, Setting and Population

**Figure 1:**
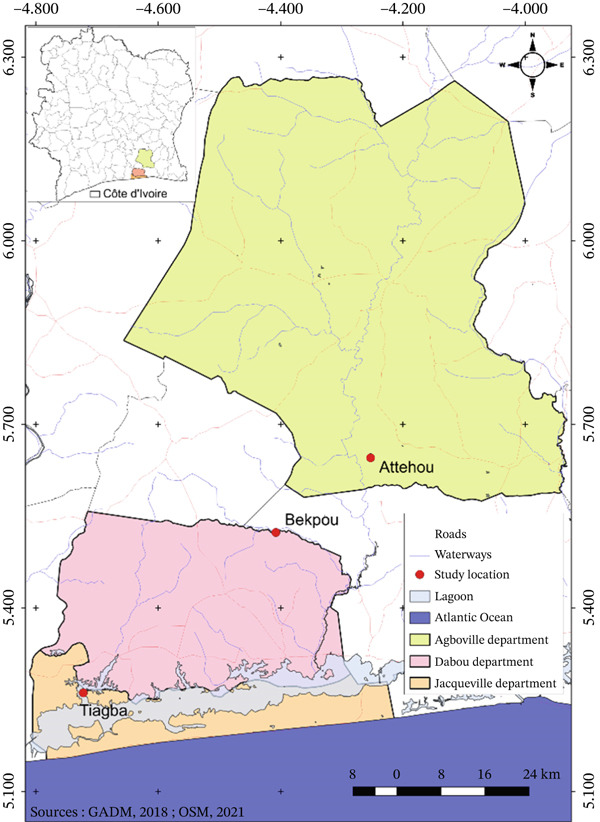
Map of the three study sites in the health districts of Dabou, Jacqueville and Agboville, southern Côte d′Ivoire. The map was created using a basemap shapefile from the Database of Global Administrative Areas (GADM [https://gadm.org/%5d; license: https://gadm.org/license.html) and OSM (2021).

The study protocol received approval from the *Comité National d′Éthique des Sciences de la Vie et de la Santé* of Côte d′Ivoire. A cross‐sectional study was conducted in October 2024 in Attehou, Bekpou and Tiagba in southern Côte d′Ivoire to investigate the relationship between environmental contamination (soil and water) and the prevalence of STH infections in humans and animals. The three villages are located in the health districts of Agboville, Dabou and Jacqueville, respectively, in southern Côte d′Ivoire (Figure 1). The area is characterised by a humid tropical climate, where STH infections are endemic [26]. The main livelihoods include subsistence agriculture, fishing and cash crops production, particularly cocoa, oil palm and rubber [11]. Open defecation is common, and domestic waste and human faeces are frequently discharged into the immediate environment [16], including rivers and lagoons shared by these villages. Access to safe drinking water remains limited for most households in these health districts [17, 18].

The study population included children aged 5 up to 12 years and livestock living in proximity to households. Soil samples were collected from children′s play areas, including household yards, public spaces and soccer fields.

## 3. Sampling Procedures

### 3.1. Soil and Water Sample Collection

For the environmental sampling, each village was subdivided into four (04) zones, within which sampling points were identified and georeferenced. These sites corresponded to locations frequently used by children, including roadsides, public gathering areas, household yards and playgrounds used as soccer fields. The number of soil samples ranged from 25 to 30 per village, and water samples from 10 to 11 per village.

Soil samples were collected from the surface layer after removing leaves and other debris. Approximately 500 g of soil was collected at each site using a plastic spatula and stored in labelled plastic bags indicating the sample identification number, date and location. To prevent cross‐contamination, the spatula was changed between sampling sites. Soil samples were collected during the rainy season and restricted to the surface layer (0–3 cm), where children are most likely to come into contact [14]. Previous studies have also shown that *Ascaris* and *Trichuris* eggs are mainly concentrated at this depth [27].

Water sampling sites were selected using a risk‐based approach, targeting locations with a high probability of faecal contamination, including human–water contact points, washing areas, animal access points and sites receiving runoff or wastewater discharges. This approach is consistent with the recommended environmental surveillance strategies for STHs and water, sanitation and hygiene (WASH) assessments [28, 29]. Water samples were collected at a depth of approximately 10–30 cm below the surface using sterilised 1‐L plastic bottles. Each bottle was labelled with the village name, date and sampling point. Two samples were collected per site as a backup.

All soil and water samples were transported on the same day to the laboratory of the *Centre Suisse de Recherches Scientifiques* in Côte d′Ivoire (CSRS) for analysis.

### 3.2. Stool Sample Collection in Humans

Written informed consent was obtained from parents or guardians of school‐age children, and assent was obtained from the children. Each consenting participant received a prelabelled 125‐mL plastic container the day before stool sample collection and was instructed to provide a fresh stool sample the following morning. Samples were collected between 6:00 and 8:00 by trained community health workers (CHWs). Between 8:00 and 10:00, sample identifiers were verified against the recruitment log, and remaining samples were processed. Transport time from collection sites to the laboratory was approximately 1 h. Upon arrival, samples were processed immediately, and the total time between collection and laboratory processing did not exceed 5 h. Samples from Dabou and Jacqueville health districts were analysed at *Hôpital Méthodiste* of Dabou (HMD), whereas those from Agboville were processed at *Centre de Santé Urbain* (CSU) of Azaguié.

### 3.3. Stool Sample Collection in Animals

In the study area, livestock are mainly reared under extensive household farming systems. As animals move freely, a wide‐meshed net was installed in frequently visited areas to create a temporary enclosure. Animals were herded into the enclosure with the assistance of four trained community members. For pig sampling, a lasso was used to restrain them safely and prevent injury. A veterinary technician equipped with gloves, protective clothing, boots and a mask collected faecal samples directly from the rectum to avoid contamination. After sampling, animals were marked with coloured paint to prevent resampling. Only accessible animals were included. Each sample was individually labelled and transported to the district laboratory for parasitological examination.

## 4. Laboratory Procedures

### 4.1. Soil and Water Samples

Water samples (1 L) were allowed to sediment for 24 h, after which the supernatant was discarded, leaving a 50‐mL pellet. The pellet was homogenised, and 10 mL was taken for analysis. An aliquot (10 mL) was preserved in sodium acetate‐acetic acid‐formalin (SAF) solution. The suspension was centrifuged at 1500 rpm for 5 min. After discarding the supernatant, 3 mL of ether was added, followed by centrifugation at 1500 rpm for 3 min. The pellet was examined microscopically by placing 100 *μ*L between a slide and a coverslip at 10x and 40x magnification, and helminth eggs were counted and recorded.

For soil samples, an optimised sieve‐flotation technique was used to detect and quantify STH eggs and larvae. Samples were air‐dried for 24 h. Ten grams (10 g) of dried soil were sieved through a series of decreasing mesh sizes (500, 200, 90, 50 and 25 *μ*m) to remove debris and concentrate eggs. The 50–25‐*μ*m fraction was collected in a 15‐mL tube, mixed with 10 mL of distilled water, and centrifuged at 1500 rpm for 5 min. The supernatant was discarded, and flotation solution (sodium chloride [NaCl], MgSO_4_ and saccharose) was added until a convex meniscus was formed. Each solution was tested separately. After 10–15 min, a coverslip was placed on top, then transferred to a slide for microscopic examination at 10x or 40x magnification. The detailed protocol is provided in File S1.

### 4.2. Human Stool Samples

Each stool sample was examined using a duplicate Kato–Katz thick smear under a light microscope for identification and quantification of helminth eggs as described elsewhere [25]. Eggs were counted and recorded separately. For quality control, 10% of the slides were randomly selected and re‐examined by a third technician on the same day. In case of discrepancy, a consensus reading was performed.

### 4.3. Animal Faecal Samples

Animal faecal samples were analysed using the McMaster flotation technique [30, 31]. Approximately 3 g of faeces were homogenised in 28 mL of distilled water and filtered to remove solid debris. The filtrate was centrifuged at 1500 rpm for 3 min. The supernatant was discarded, and 7 mL of saturated NaCl solution was added to the pellet. The mixture was loaded into both chambers of a McMaster slide using a pipette, avoiding air bubbles. After 1–2 min, the slide was examined under a light microscope at 10x and 40x magnification, and eggs within the counting grids were counted. The total number of egg count per slide was multiplied by the correction factor 50 to obtain eggs per gram of faeces.

## 5. Data Analysis

Data were double entered into Microsoft Excel 2013 and checked for consistency using EpiInfo Version 3.5.4 (Centers for Disease Control and Prevention, Atlanta, Georgia, United States). Statistical analyses were performed using STATA Version 14.2 (Stata Corp, College Station, Texas, United States).

Qualitative variables were expressed as proportions with 95% confidence intervals (95% CI), quantitative variables as geometric means. Differences between proportions were assessed using Fisher′s exact test.

Associations between soil contamination with helminth eggs and infection in children were assessed using logistic regression models and expressed as odds ratios (ORs) with 95% CIs. Infection intensity, expressed as eggs per gram of stool (EPG), was analysed using negative binomial regression models and expressed as incidence rate ratios (IRRs) with 95% CIs. Multivariable models were adjusted for age and sex. Statistical significance was set at *p* < 0.05.

## 6. Results

### 6.1. Characteristics of the Study Populations

A total of 215 stool samples from school‐aged children, 58 from livestock and 127 environmental samples (41 stagnant water and 86 soil samples from playgrounds) were collected. More than half of the children were girls (54.41%). The mean age was 8.7 years (ranged from 5 to 12 years). Among the 58 animals sampled, 9 (15.51%) were pigs, 15 (25.86%) were goats, and 34 (58.62%) were sheep. Most sheep were sampled in Attehou (22/34) (Table 1).

**Table 1:** Characteristics of the human and animal populations in the three investigated villages in southern Côte d′Ivoire (October 2024).

Characteristics	Attehou	Bekpou	Tiagba	*n*(%)
Humans (sex)				
Male	35	41	22	98 (45.58)
Female	34	56	27	117 (54.41)
Total	69	97	49	215
Humans (age groups)				
5–9 years	40	62	32	134 (62.32)
10–12 years	29	35	17	81 (37.67)
Total	69	97	49	215
Animals (type)				
Pig	2	4	3	9 (15.51)
Goat	4	11	0	15 (25.86)
Sheep	22	9	3	34 (58.62)
Total	28	24	6	58
Environment (sample)				
Soil	35	25	26	86 (67.72)
Water	16	14	11	41 (32.28)
Total	51	39	37	127

### 6.2. Prevalence of STH Across the Investigated Matrices

**Figure 2:**
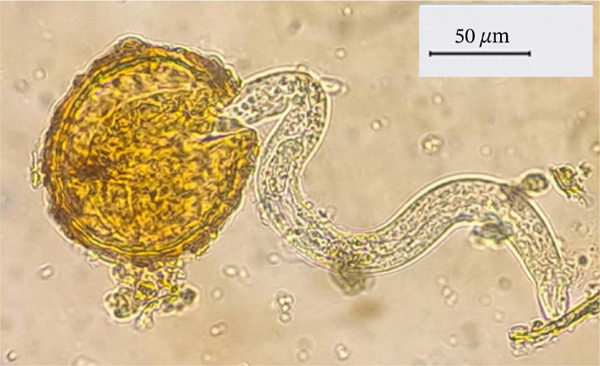
Viable *Ascaris* egg in a soil sample taken from the village of Attehou in southern Côte d′Ivoire (October 2024). This figure shows a viable *Ascaris* egg releasing an infective larva, image taken in the laboratory of the Centre Suisse de Recherches Scientifiques en Côte d′Ivoire (CSRS) during microscopic observation (objective ×40, eyepiece ×10, total magnification ×400) after the flotation technique with saturated NaCl as the flotation solution (January 2025). Credit photo: Touré Sadikou, Senior Technician at CSRS.

The overall prevalence of soil‐transmitted infections was 44.6% (95% CI: 37.8–51.5) in children and 24.1% (95% CI: 13.8–37.1) in livestock. In environmental samples, prevalence was 25.5% (95% CI: 16.7–36.1) in soil and 2.4% (95% CI: 0.1–12.8) in water. The parasites identified were *Ascaris*, *Trichuris* and hookworms. *Trichuris* was the most frequently detected parasite in faecal samples (24.7%; 95% CI: 20.0–29.2), particularly among children, whereas *Ascaris* showed the highest prevalence in soil (24.4%). Hookworm larvae were rare and detected only in water samples (2.4%; 95% CI: 0.1–12.8) (Table 2). A viable *Ascaris* egg with a developing larva was observed in a soil sample from Attehou during laboratory examination (Figure 2).

**Table 2:** Distribution of helminth eggs in humans, animals′ water and soil, in three villages in southern Côte d′Ivoire in October 2024.

	Stagnant water	Soil	Animals^a^	School‐aged Children	Total	
	(*N* = 41)	(*N* = 86)	(*N* = 58)	(*N* = 215)	(*N* = 400)	
Parasite species	*n*(%)	*n*(%)	*n*(%)	*n*(%)	*n*(%)	95% CI
Hookworms	1 (2.43)	0 (0)	3 (5.1)	1 (0.4)	5 (1.2)	0.4–2.8
*Ascaris*	0 (0)	21 (24.4)	5 (8.62)	34 (15.8)	60 (15)	11.6–18.8
*Trichuris*	0 (0)	4 (4.6)	10 (17.2)	85 (39.5)	99 (24.7)	20.5–29.2
Helminth	1 (2.43)	22 (25.5)	14 (24.1)	96 (44.6)	133 (33.2)	28.6–38.1

^a^Animals were sheep, goats and pigs; *N*: total number of samples, *n*: parasite number and %: prevalence.

**Figure 3:**
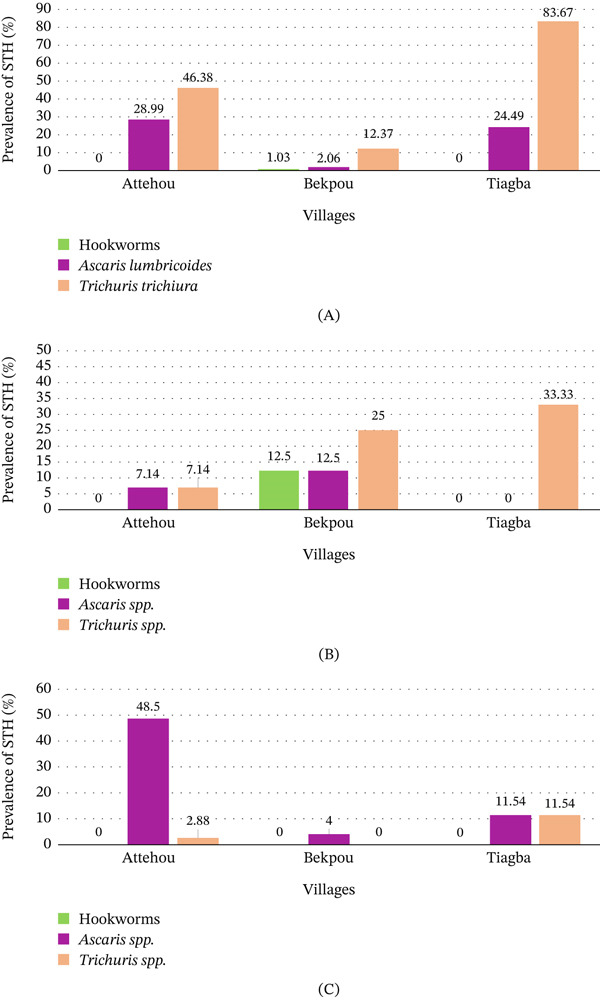
Prevalence of STH (A) in school‐age children, (B) in animals and (C) in soil samples collected in the villages of Attehou, Bekpou and Tiagba, Southern Côte d′Ivoire, in October 2024.

Among children, the prevalence of *T. trichiura* and *A. lumbricoides* varied by village, ranging from 28.9% (95% CI: 18.6–41.1) to 83.6% (95% CI: 70.3–92.6). *T. trichiura* infection was the highest in Tiagba, followed by Attehou (83.6%, 95% CI: 70.3–92.6 and 46.3%, 95% CI: 34.2–58.7, respectively), whereas *A. lumbricoides* was most prevalent in Attehou (28.9%, 95% CI: 18.6–41.1). A hookworm larva was identified in Bekpou, corresponding to a prevalence of 1.03% (95% CI: 0.1–5.6) (Figure 3A).

Of the 58 animals screened, 14 were infected with at least one STH species (24.1%, 95% CI: 13.8–37.1). *Trichuris* spp. was predominant, with a prevalence of 33.3% (95% CI: 4.3–77.7) in Tiagba and 25% (95% CI: 9.7–46.7) in Bekpou. Hookworms were detected only in Bekpou (12.5%, 95% CI: 2.6–32.3) (Figure 3B).

Of the 86 soil samples, 22 were positive for helminth eggs (25.5%; 95% CI: 16.7–36.1). Attehou showed the highest soil contamination with *Ascaris* spp. (48.5%; 95% CI: 31.3–66.1), followed by Tiagba, where *Ascaris* spp. and *Trichuris* spp. were detected at similar levels (11.54%; 95% CI: 2.4–30.1). No hookworm larvae were detected in soil samples (Figure 3C).

A total of 172 helminth eggs were recovered from soil samples. Attehou accounted for the highest egg burden (*n* = 109; 63.3%; 95% CI: 55.6–70.5), predominantly *Ascaris* (107 eggs; 66.4%; 95% CI: 58.6–73.6), followed by Tiagba (61 eggs; 35.4%; 95% CI: 28.3–43.1), including 52 *Ascaris* and 9 *Trichuris* eggs. Bekpou showed the lowest egg burden, with only 2 *Ascaris* eggs (1.2%; 95% CI: 0.1–4.4). Overall, a heterogeneous distribution of helminth eggs was observed across matrices and villages (Table 3).

**Table 3:** Number of helminth eggs found in soil samples by village in southern Côte d′Ivoire, October 2024.

	Attehou		Bekpou		Tiagba	
Parasite species	Nber of eggs	% (95% CI)	Nber of eggs	% (95% CI)	Nber of eggs	% (95% CI)
*Ascaris*	107	66.4 (58.6–73.6)	2	1.2 (0.1–4.4)	52	32.2 (25.1–40.1)
*Trichuris*	2	18.1 (2.2–51.7)	0	00 (n.a.)	9	81.18 (48.2–97.7)
Hookworms	0	00 (n.a.)	0	00 (n.a.)	0	00 (n.a.)
Total	109	63.3 (55.6–70.5)	2	1.2 (0.1–4.4)	61	35.4 (28.3–43.1)

*Note:* n.a.: not applicable; %: prevalence; Nber of eggs: number of eggs.

### 6.3. Pattern of Eggs′ Abundance in the Four Matrices Investigated (Stagnant Water, Soil, Human and Animal)

Higher soil contamination with helminth eggs was associated with increased risk of infection in children. For *Trichuris* infection, children living in areas with intermediate and high contamination levels had 5.94‐fold (95% CI: 5.90–5.98; *p* < 0.001) and 38.39‐fold (95% CI: 32.54–45.30; *p* < 0.001) higher odds of infection, respectively, compared with those in low‐contamination areas. Increased soil contamination was also significantly associated with higher infection intensity (IRR = 6.11; 95% CI: 1.92–19.46; *p* = 0.002), indicating a consistent relationship between environmental contamination and parasite burden (Tables 4 and 5).

**Table 4:** Factors associated with parasite burden (eggs per gram) of *Trichuris trichiura* and *Ascaris lumbricoides* in children and contaminated soil samples using a negative binomial regression with robust standard errors adjusted for village clustering (*N* = 215).

	*Trichuris trichiura* (EPG)			*Ascaris lumbricoides* (EPG)		
Variables	IRR adjusted	95% CI	*p*	IRR adjusted	95% CI	*p*
Soil contamination						
Number of eggs in the soil	6.11	1.92–19.46	0.002	2.98	0.48–18.58	0.242
Sex						
Male	1			1		
Female	4.18	3.68–4.75	< 0.001	0.78	0.69–0.87	< 0.001
Age (years)						
5–9	1			1		
10–15	1.25	0.72–2.19	0.431	6.12	1.92–19.54	0.002

Abbreviations: 95% CI, 95% confidence interval; EPG, eggs per gram of stool; IRR, adjusted incidence rate ratio.

**Table 5:** Assessment of factors associated with *Trichuris trichiura* and *Ascaris lumbricoides* infections in children and contaminated soil samples using a multivariate logistic regression with robust standard errors adjusted for village clustering (*n* = 3 clusters).

	*Trichuris trichiura* infection (*N* = 215)			*Ascaris lumbricoides* infection (*N* = 215)		
Variable	OR adjusted	95% CI	*p*	OR adjusted	95% CI	*p*
Soil contamination						
Low	1			1		
Moderate	5.94	5.90–5.98	< 0.001	15.55	15.01–16.11	< 0.001
High	38.39	32.54–45.30	< 0.001	19.68	17.80–21.76	< 0.001
Sex						
Male	1			1		
Female	1.55	0.90–2.68	0.116	0.81	0.36–1.83	0.615
Age (years)						
5–9	1			1		
10–15	1.53	0.71–3.29	0.278	1.09	0.71–1.68	0.691
Pseudo − *R* ^2^	0.28			0.16		

*Note:* Standard errors are adjusted for intravillage correlation (3 clusters). Low, < 1 egg/g of soil; Moderate, 1–5 eggs/g of soil; High, > 5 eggs/g of soil.

Abbreviations: 95% CI, 95% confidence interval; OR, adjusted odds ratio.

For *Ascaris* infection, children living in areas with intermediate and high contamination levels also had a higher risk of infection, with adjusted ORs of 15.55 (95% CI: 15.01–16.11; *p* < 0.001) and 19.68 (95% CI: 17.80–21.76; *p* < 0.001), respectively. However, no significant association was observed between soil contamination and infection intensity (IRR = 2.98; 95% CI: 0.48–18.58; *p* = 0.242) (Tables 4 and 5).

Overall, these findings highlight the key role of soil contamination in the transmission of helminth infections, with a stronger and more consistent association observed for *T*. *trichiura* than for *A*. *lumbricoides*.

### 6.4. Performance of Flotation Solutions in the Identification of STH Eggs

The number of *Ascaris* and *Trichuris* eggs recovered varied according to the flotation solution used. Saturated NaCl solution yielded the highest number of *Ascaris* eggs, accounting for 75.15% (121/161) of all eggs detected, followed by SAF (15.53%). Although fewer *Trichuris* eggs were detected overall, a similar pattern was observed across flotation solutions (Table 6).

**Table 6:** Recovery rate of the most detected helminth (*Ascaris* and *Trichuris*) eggs in soil samples according to the flotation solution.

Type of solutions	*Ascaris* spp.		*Trichuris* spp.	
	Number of eggs detected (%)	*p* value	Number of eggs detected (%)	*p* value
NaCl	121 (75.15)		5 (45.45)	
MgSo_4_	15 (9.32)		1 (9.09)	
Saccharose	0 (0.00)		0 (0.00)	
SAF	25 (15.53)		5 (45.45)	
Total	161 (100)	< 0.001 ^∗^	11 (100)	0.089 ^∗^

^∗^ *p* value was assessed using Fisher′s exact test.

## 7. Discussion

Environmental contamination by STH eggs poses a risk to human and animal health. The eggs of parasites such as *Ascaris* spp. and *Trichuris* spp. are highly resistant and can survive in water and soil for several months or even years [25, 32]. This study assessed environmental contamination by STH eggs in soil, stagnant water and livestock to evaluate whether environmental contamination could serve as a proxy for human or animal parasitological screening for the rapid identification of high‐risk communities. Moderate to low levels of contamination were detected across the matrices investigated, with marked differences between sample types and study sites. The highest prevalence was observed in school‐aged children (44.6%; 97/215), followed by soil samples (25.5%; 22/86) and livestock (24.1%; 14/58), whereas stagnant water showed very low contamination (2.43%; 1/41) [33].

The high prevalence observed among school‐aged children is consistent with existing literature showing that this age range is particularly vulnerable to STH infections due to frequent exposure‐related behaviours, namely inadequate hygiene practices and regular contact with contaminated environments, especially in rural areas [34–36].

Soil contamination, with more than one‐quarter of samples testing positive, reflects substantial environmental exposure to STHs. The detection of helminth eggs in pigs, sheep and goats likely reflects shared exposure to contaminated environments rather than a major contribution of livestock to human transmission. Most human STHs are primarily anthroponotic and maintained through human faecal contamination of the environment [7, 37]. Although occasional zoonotic transmission may occur, particularly with *A*. *suum*, livestock are generally not considered major reservoirs of human infection [2, 38]. These findings therefore point out inadequate sanitation and environmental hygiene as the main drivers of STH transmission.

Stagnant water was the least contaminated matrix across all villages. This contrasts with findings from studies conducted in Ethiopia that reported higher levels of helminth egg contamination in wastewater [25, 39]. Differences in contamination levels may be explained by the nature of the sampled water bodies, with Ethiopian studies focusing on heavily polluted wastewater systems, whereas our study targeted stagnant surface water.

The distribution of helminth species varied across matrices. *Ascaris* spp. predominated in soil, whereas *Trichuris* spp. were more frequently detected in children and livestock. The predominance of *Ascaris* in soil is consistent with the high resistance of its eggs, which can remain viable for several months under favourable conditions [37]. At the village level, Attehou showed the highest soil egg burden. This may partly reflect differences in soil characteristics, as sandy soils facilitate egg recovery during flotation procedures more than clay‐rich soils [24, 40, 41].

Higher soil contamination was associated with higher levels of infection in children, consistent with previous studies highlighting the importance of contaminated soil in the transmission of STHs [42, 43].

Several flotation solutions were evaluated as affordable alternatives to molecular diagnostics for environmental surveillance. Saturated NaCl solution yielded the highest number of *Ascaris* and *Trichuris* eggs. Its density is well suited to the flotation of STH eggs and offers a practical balance between performance, cost and ease of use compared with zinc sulphate and sucrose solutions [44, 45]. These findings highlight the importance of selecting flotation solutions according to the target helminth species. The detection of STH eggs in all investigated matrices highlights the widespread environmental contamination and the continuous exposure of humans and animals to STH infections [46, 47]. These findings support the implementation of integrated STH control strategies based on a One Health approach [48, 49].

This study has several limitations. Firstly, the lack of molecular confirmation (PCR), particularly for environmental samples, may have limited the accurate identification of parasitic species. Future studies incorporating molecular techniques would improve diagnostic accuracy and strengthen the assessment of environmental transmission. Furthermore, this study was not designed to assess the zoonotic transmission route between humans and animals. Longitudinal or experimental studies would be necessary to establish a causal link, particularly through comparative molecular and genetic analyses. Secondly, the sample sizes for animal, soil and water samples were based on case availability, which may have introduced sampling bias and led to an underestimation of prevalence across these environmental matrices. Thirdly, different diagnostic approaches were used depending on the type of sample, each with varying levels of sensitivity and specificity. This methodological heterogeneity could limit the direct comparability of results across matrices.

## 8. Conclusion

The prevalence of STH eggs in soil alone does not appear sufficient to identify high‐risk communities. However, soil egg density may provide a more informative indicator of transmission intensity in endemic settings. In areas implementing WASH interventions, environmental monitoring of helminth eggs may also contribute to evaluating progress towards reducing open defecation and interrupting transmission. The modified flotation technique used in this study represents a simple and affordable approach for environmental surveillance in resource‐limited settings where molecular tools remain largely inaccessible. Further studies are needed to validate this approach across different environmental and epidemiological contexts. Overall, our findings support the integration of environmental surveillance within a One Health framework for strengthening STH control and elimination efforts.

**Nomenclature**
CSU: Centre de Santé Urbain
FS: flotation solution
HMD: Hôpital Méthodiste de Dabou
STH: soil‐transmitted helminth
WHO: World Health Organisation

## Author Contributions

Contribution to study design: N.A.K. and J.T.C.; investigation: N.A.K., T.J.D.A, M‐J.A.A‐B., S.T., L.K.V., K.D.S. and J.T.C.; statistical analysis and writing of the manuscript: N.A.K., J.N.K., and J.T.C.; all authors contributed to the revision of the manuscript.

## Funding

No funding was received for this manuscript.

## Disclosure

All authors read and approved the submitted version.

## Ethics Statement

This study was approved by the *Comité National d′Éthique des Sciences de la Vie et de la Santé* of Côte d′Ivoire (Reference: 018‐23/MSHPCMU/CNESVS‐km) and conducted in accordance with the ethical principles of the current version of the Declaration of Helsinki (ICH GCP Guidelines E6/R2). Written consent was obtained from parents or guardians of participating children, and written assent was obtained from the children. Participation was voluntary, and participants were free to withdraw from the study at any time. All children infected with STHs received a single oral dose of 400‐mg albendazole. As Côte d′Ivoire does not have a dedicated ethics committee for animal research, animal procedures were conducted in accordance with the principles of the Basel Declaration. Faecal samples were collected by an experienced veterinary technician with the consent of the animal owners and in a manner that minimised animal discomfort.

## Consent

The authors have nothing to report.

## Conflicts of Interest

The authors declare no conflicts of interest.

## Supporting information


**Supporting Information** Additional supporting information can be found online in the Supporting Information section. Supporting Information. File S1: The standard operating procedure (SOP) describing the methodology used for the collection, processing, and analysis of environmental samples for soil‐transmitted helminth egg detection.

## Data Availability

The data that support the findings of this study are available on request from the corresponding author. The data are not publicly available due to privacy or ethical restrictions.
