# Supplementary material for: Assessment of Environmental Contamination With Soil‐Transmitted Helminth Eggs From Human and Animal Faeces in Southern Côte d′Ivoire: A Rapid Approach for Identifying High‐Risk Communities
Source: J Parasitol Res. 2026 Jul 24;2026:2672432. doi: 10.1155/japr/2672432 (PMC13400998; doi:10.1155/japr/2672432)
Supplement: Supplementary file 1 — Supporting Information Additional supporting information can be found online in the Supporting Information section. Supporting Information. File S1: The standard operating procedure (SOP) describing the methodology used for the collection, processing, and analysis of environmental samples for soil‐transmitted helminth egg detection. [file JAPR-2026-2672432-s001.docx]

**Standard Operating Procedure (SOP)**

**Recovery and identification of soil-transmitted helminth (STH) eggs in environmental soil samples**

**1- Scientific justification**

Soil contamination by soil-transmitted helminths (STHs) is a key indicator of parasite transmission in the environment [1]**.** However, the diversity of protocols aiming to assess soil-contamination by soil-transmitted helminths is making hard studies comparability and reproducibility [2,3]. Therefore, a standardised procedure is required to ensure reproducibility and comparability between studies.

This procedure follows the environmental surveillance guidelines proposed by the World Health Organisation for assessing risks related to sanitation and environmental contamination by faeces, which ensures a sustained transmission of STHs [4]

22**2- Scope**

This SOP applies to soil samples collected from a variety of sites in each location, namely, domestic properties, schools, community spaces, areas adjacent to domestic properties, and areas that are regularly damp or retain moisture, to assess environmental contamination by STH eggs.

**3- Overview of the method**

Sampling → Sieving → Sedimentation / Flotation → Microscopy → Quantification

- **Sampling**
- Collect soil samples from the surface layer (0-3 cm).
- Remove the leaf litter.
- Use approximately 500 g of soil per collection site using a plastic spatula, which is replaced between each site to prevent cross-contamination
- Labelled bags and stored in a dedicated, uncontaminated storage area
- It is important to ensure that only the surface layer is collected, as it represents the layer with which people are most experienced.

**4- Equipment**

- Precision balance (±0.01 g)
- Mesh sieves (500 µm, 200 µm, 90 µm, 50 µm, and 25 µm)
- Centrifuge (1500–2000 rpm)
- Optical microscope

**5- Consumables**

- 15 mL and 50 mL conical tubes
- Pasteur pipettes
- Beakers
- Slides and cover slips

**6- Reagents**

| **Reagents** | **Specification** |
| --- | --- |
| Distilled water | **N/A** |
| Magnesium sulphate (MgSO₄) | Density 1.20 |
| Sodium chloride (NaCl) | Density 1.20 |
| Sucrose | Density 1.27 |
| Sodium acetate–acetic acid–formalin (SAF) | **N/A** |

N/A, Not Applicable

**7- Analytical procedure**

**With flotation solutions** (NaCl, MgSO₄, sucrose)

1- Homogenise the soil sample

2- Weigh out 10 g of soil

3- Sieve the 10 g of soil respectively, through sieves with mesh sizes of 500 µm, 200 µm, 90 µm, 50 µm, and 25 µm

4- Collect the material retained on the 50 µm and 25 µm sieves into a 15 ml tube

5- Add 10 ml of distilled water

6- Centrifuge at 1,500 rpm for 5 minutes

7- Remove the supernatant

8- Add a flotation solution (NaCl, MgSO₄, sucrose) until a convex meniscus forms (repeat separately for each solution)

9- Cover with a coverslip for 10 minutes and leave to stand for 10 to 15 minutes to allow the eggs and larvae to float and adhere to the coverslip

10- Place the smear on a slide.

**With SAF**

1- Weigh 5 g of soil into a 50 mL conical flask.

2- Add 20 mL of SAF solution (sodium acetate–acetic acid–formalin).

3- Mix vigorously for 1 to 2 minutes

4- Filter the suspension through a double-layered piece of gauze into a new 50 mL

tube to remove coarse particles.

5- Rinse the residue retained on the gauze with 5 mL of SAF

6- Centrifuge the filtrate at 1,500 rpm for 5 minutes.

7- Carefully remove the supernatant without disturbing the pellet.

8- Resuspend the pellet in 10 mL of SAF.

9- Add 3 mL of ethyl acetate.

10-Shake vigorously for 30 seconds.

11- Centrifuge at 1,500 rpm for 10 minutes

12- After centrifugation, remove the upper layers (ethyl acetate, debris and liquid).

13- Retain only the pellet at the bottom of the tube.

14- Resuspend the pellet in 1 to 2 mL of SAF

15- Place 50 µL of sediment onto a slide and cover it with a coverslip

- **Microscopic observation**

Examine the entire surface systematically:

• ×10 objective: screening

• ×40 objective: morphological confirmation

**8- Criteria for identifying parasites**

Identification based on standard morphological keys:

| **Parasite** | **Features** |
| --- | --- |
| *Ascaris* spp. | Thick, bumpy shell |
| *Trichuris* spp. | Polar caps |
| Hookworms | Thin shell, segmented embryo |

**9- Presentation of results**

The results are reported as:

• Presence/absence,

• Number of eggs per 10 grams of soil

**10- Quality assurance**

• Duplicate analysis of 10% of samples.

• Independent double-reading.

• Positive control slides are used regularly.

• Decontamination of equipment between samples.

**11- Biosafety**

**•** Personal protective equipment (PPE) must be worn (gloves, lab coat).

• Surfaces must be disinfected after handling.

• Biological waste must be disposed of in accordance with biosafety standards.

**12- Limitations of the method**

**•** Spatial heterogeneity of soil contamination.

• Potential reduction in yield in clay soils.

• Aggregation of eggs affects quantification.

Haut du formulaire

**Reference**

1. WHO. Soil-transmitted helminth infections [Internet]. 2023 [cited 2025 Apr 10]. https://www.who.int/news-room/fact-sheets/detail/soil-transmitted-helminth-infections. Accessed 10 Apr 2025

2. Khurana S, Singh S, Mewara A. Diagnostic Techniques for Soil-Transmitted Helminths - Recent Advances. Res Rep Trop Med. 2021;12:181–96. https://doi.org/10.2147/RRTM.S278140

3. Manuel M, Amato HK, Pilotte N, Chieng B, Araka SB, Siko JEE, et al. Soil surveillance for monitoring soil-transmitted helminth infections: method development and field testing in three countries [Internet]. medRxiv; 2023 [cited 2026 June 1]. p. 2023.09.26.23296174. https://doi.org/10.1101/2023.09.26.23296174

4. WHO. Wastewater and environmental surveillance for one or more pathogens: guidance on prioritization, implementation and integration [Internet]. 2024 [cited 2026 May 2]. https://www.who.int/publications/m/item/wastewater-and-environmental-surveillance-for-one-or-more-pathogens--guidance-on-prioritization--implementation-and-integration. Accessed 2 May 2026
